# Phylogenetic Analysis of the 2020 West Nile Virus (WNV) Outbreak in Andalusia (Spain)

**DOI:** 10.3390/v13050836

**Published:** 2021-05-05

**Authors:** Carlos S. Casimiro-Soriguer, Javier Perez-Florido, Jose L. Fernandez-Rueda, Irene Pedrosa-Corral, Vicente Guillot-Sulay, Nicola Lorusso, Luis Javier Martinez-Gonzalez, Jose M. Navarro-Marí, Joaquin Dopazo, Sara Sanbonmatsu-Gámez

**Affiliations:** 1 Clinical Bioinformatics Area, Fundación Progreso y Salud (FPS), Hospital Virgen del Rocio, 41013 Sevilla, Spain; carlos.sanchez.c@juntadeandalucia.es (C.S.C.-S.); javier.perez.florido.sspa@juntadeandalucia.es (J.P.-F.); josel.fernandez.rueda@juntadeandalucia.es (J.L.F.-R.); 2 Computational Systems Medicine, Institute of Biomedicine of Seville (IBIS), Hospital Virgen del Rocio, 41013 Sevilla, Spain; 3 Laboratorio de Referencia de Virus de Andalucía, Servicio de Microbiología, Hospital Virgen de las Nieves, 18014 Granada, Spain; irenee.pedrosa.sspa@juntadeandalucia.es (I.P.-C.); vicentel.guillot.sspa@juntadeandalucia.es (V.G.-S.); josem.navarro.sspa@juntadeandalucia.es (J.M.N.-M.); 4 Instituto de Investigación Biosanitaria, ibs.GRANADA, 18012 Granada, Spain; 5 Dirección General de Salud Pública y Ordenación Farmacéutica, Consejería de Salud y Familias, Junta de Andalucía, 41020, Sevilla, Spain; nicola.lorusso.sspa@juntadeandalucia.es; 6 GENYO, Centre for Genomics and Oncological Research: Pfizer—University of Granada—Andalusian Regional Government, 18016 Granada, Spain; luisjavier.martinez@genyo.es; 7 Bioinformatics in Rare Diseases (BiER), Centro de Investigación Biomédica en Red de Enfermedades Raras (CIBERER), FPS, Hospital Virgen del Rocio, 41013 Sevilla, Spain; 8 ELIXIR.ES/FPS, Hospital Virgen del Rocio, 41013 Sevilla, Spain

**Keywords:** West Nile Virus, outbreak, meningoencephalitis, epidemiology, phylogeny, whole genome sequencing

## Abstract

During recent decades West Nile Virus (WNV) outbreaks have continuously occurred in the Mediterranean area. In August 2020 a new WNV outbreak affected 71 people with meningoencephalitis in Andalusia and six more cases were detected in Extremadura (south-west of Spain), causing a total of eight deaths. The whole genomes of four viruses were obtained and phylogenetically analyzed in the context of recent outbreaks. The Andalusian viral samples belonged to lineage 1 and were relatively similar to those of previous outbreaks which occurred in the Mediterranean region. Here we present a detailed analysis of the outbreak, including an extensive phylogenetic study. As part on this effort, we implemented a local Nextstrain server, which has become a constituent piece of regional epidemiological surveillance, wherein forthcoming genomes of environmental samples or, eventually, future outbreaks, will be included.

## 1. Introduction

West Nile virus (WNV), a member of the Flavivirus genus, is transmitted in an enzootic cycle involving birds as amplifying hosts and mosquitoes as vectors [1], which can ultimately be transmitted to mammals, considered dead-end hosts, causing disease outbreaks in horses and/or humans [2]. Currently, the virus is considered a recurrent zoonosis with a wide geographic distribution [3]. Phylogenetically, WNV is classified into eight lineages [4], although highly pathogenic strains belong mainly to lineages 1 and 2 [5]. There is evidence of WNV circulation in Europe since the 1950s [6], and the first recognized outbreak in humans occurred in 1962 in southern France [7]. Lineage 1 was identified in the majority of outbreaks in horses and humans in Europe [8], until 2004 when lineage 2 was detected for the first time in Hungary [9]. In 2004, in addition (and simultaneously) to the introduction of lineage 2 WNV in Hungary, there was another independent introduction in Southern Russia that has also spread from there since [10]. By 2008, lineage 2 had spread through Central Europe, Russia, and the Eastern Mediterranean basin, where the virus has remained endemic. Both lineages coexist in countries like Italy, where outbreaks have been reported every year since 2008 [11], including WNV from lineages 1 and 2 [12], and also in Cyprus by 2016 [13], as well as in other countries like Greece, Serbia, etc. In Spain, WNV lineage 1 was responsible for the outbreaks which occurred in 2008, 2010, and 2016 [14,15], while the presence of lineage 2 was recently confirmed in birds in Catalonia [16]. In 2020, European Union states declared 316 locally acquired human cases of WNV infections, most of them reported by Greece (143), Spain (77), and Italy (66). Eight provinces in Bulgaria, Spain, The Netherlands, and Germany have declared locally acquired human cases for the first time, indicating further expansion of WNV in Europe [17].

**Figure 1:**
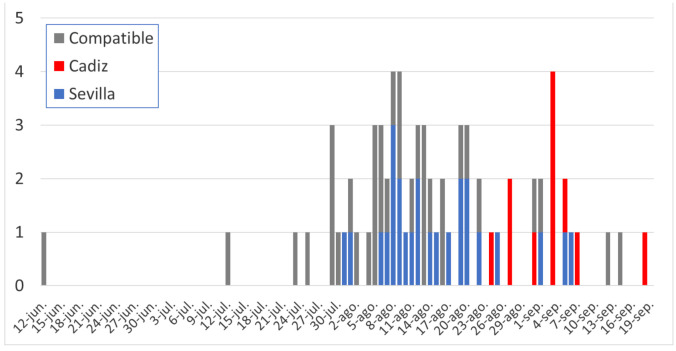
Epidemic curve of confirmed cases of WNV meningoencephalitis, by province of exposure (blue, Sevilla; red, Cádiz), as well as probable WNV cases detected by retrospective analysis (grey).

In August 2020, five West Nile Fever (WNF) human cases with unknown lymphocytic meningoencephalitis were first identified in the province of Seville (Andalusia) in two neighboring municipalities in front of the Guadalquivir marsh (Puebla del Río and Coria del Río). Slightly later, four outbreaks were reported in equine holdings in the same province. A month later, another group of human cases was identified in the province of Cádiz. The whole outbreak, as reported by the Andalusian Epidemiological Surveillance System (SVEA), comprised 71 human cases of WNV meningoencephalitis (36 confirmed cases), with eight deaths, representing an 11.3% mortality rate (Figure 1). Later, six more cases occurred in Badajoz, in the autonomous community of Extremadura, bordering Andalusia. Moreover, retrospective analysis of deaths occurring in the same localities on similar dates indicated 35 more individuals with probable WNV infection symptoms, and thus were most likely also affected by WNV (See Figure 1). Five cases that were confirmed by PCR (four from Sevilla and one from Cádiz) belonged to lineage 1 [18].

In this work we provide a detailed molecular characterization of the most recent WNV outbreak in Andalusia (Spain).

## 2. Materials and Methods

### 2.1. Samples, Molecular Diagnosis, and Culture Isolation

According to the protocol for West Nile fever surveillance and alert, clinical samples from suspected cases of human WNV neurological disease were referred to our Andalusian Virus Reference Laboratory for laboratory diagnosis and, depending on the laboratory and epidemiological criteria, the cases were classified as confirmed or probable [19]. The following diagnostic tests were performed in order to confirm WNV infection [20,21]: detection of virus-specific IgM and IgG antibodies in serum and/or cerebrospinal fluid (CSF) by ELISA testing (Euroimmun, Lübeck, Germany), and detection of specific viral nucleic acids in CSF, serum, and/or urine. Nucleic acid extraction from clinical samples was performed using a QIAsymphony DSP virus/pathogen mini kit (QIAGEN, Hilden, Germany). Real-time reverse-transcription polymerase chain reaction (qRT-PCR) targeting a conserved sequence of the 3′-untranslated (3′UTR) region of WNV genome was used to confirm the presence of specific viral RNA [22] in 6 urine samples and 1 CSF sample from 6 patients (see Table 1). For virus isolation, all of the procedures were performed within certified biosafety cabinets under biosafety level 3 (BSL3) containment. WNV RNA-positive samples were inoculated onto confluent monolayers of Vero cells. Passage to fresh Vero cell tubes was performed after 10 days of incubation or when cytopathic effect was observed [23,24]. Viral growth was confirmed by qRT-PCR [22] of the cell culture supernatant and viral culture was considered negative after 2 passages without evidence of CPE and negative qRT-PCR of the supernatant. WNV was isolated after one passage from the urine sample of one case from Cádiz. Purified RNA from qRT-PCR-positive samples with ct values <30, along with one WNV isolate, were used for sequencing.

### 2.2. Viral Sequencing 

RNA was quantified by NanoDrop (Thermo Scientific, Waltham, MA USA) and verified through use of a Bioanalyzer 2100 with RNA 6000 Nano Kit (Agilent Technologies, Santa Clara, CA, USA). SuperScript IV Reverse Transcriptase (Thermo Fisher Scientific, Waltham, MA USA) was used for cDNA synthesis with 11 µL of RNA and 1 µl of random hexamer primers. To perform multiplex PCR, the Q5 Hot Start High-Fidelity (New England Biolabs, Hitchin, Hertfordshire, UK) protocol was followed by adding the primers previously described [25]. After PCR was performed with a set of 41 primer pairs, the amplified regions were purified with Agencourt AMPure XP beads (Beckman Coulter, Nyon, Switzerland). Library preparation was performed using an Illumina DNA Prep kit, following the manufacturer’s recommendations. Samples were pooled in equal concentrations after quantification by Qubit 4 Fluorometer (Invitrogen, Waltham, MA, USA). Sequencing was carried out on a MiSeq system using a Nano v2 kit (Illumina, San Diego, CA, USA).

### 2.3. Viral Genomic Data Processing

Sequencing data (150bpx2) were analyzed using in-house scripts and the nf-core/viralrecon pipeline software [26]. Briefly, after read quality filtering, sequences for each sample were aligned to the NY99 lineage 1 WNV genome (NC_009942.1) using *bowtie 2* algorithm [27], followed by primer sequence removal and duplicate read marking using *iVar* [28] and *picard* [29] tools, respectively. Genomic variants were identified through *iVar* software, using a minimum allele frequency threshold of 0.25 for calling variants, and a filtering step to keep variants with a minimum allele frequency threshold of 0.75. Using the set of high confidence variants and the NY99 genome, a consensus genome per sample was finally built using *iVar*. 

The four WNV sequences are available in the European Nucleotide Archive (ENA) database under the project identifier PRJEB43037. 

### 2.4. Phylogenetic Analysis

A phylogenetic analysis was performed on the obtained consensus genomes in the context of a world-wide representative set of WNVs (See Table S1) using the Augur application [30], whose functionality relies on the IQ-Tree software [31]. The MAFFT program [32,33], which is one of the most sensitive multiple alignment methods [34], was utilized for the multiple alignment, using the strain NC_009942.1 as reference. The phylogenetic tree was recovered by maximum likelihood, using a general time reversible model with unequal rates and unequal base frequencies [35]. Branching date estimation was carried out with the least square dating (LSD2) method [36]. Branching point reliabilities were estimated by UFBoot, an ultrafast bootstrap approximation to assess branch supports [37]. 

The variability along the viral genomes was estimated using the Shannon entropy [38]. The non-synonymous to synonymous ratios along the viral genomes were calculated using the KaKs_Calculator [39].

The results can be viewed in the Nextstrain [40] local server, which is now part of the Andalusian Genomic Epidemiology System (SIEGA) [41].

## 3. Results

### 3.1. Sample Sequencing and Sequencing Data Collection

Although the CSF sample from patient 44025400 did not grow in culture, the urine sample from the same patient was successfully used for sequencing. Unfortunately, urine samples from patients 44013531 and 44013536 resulted in a low-coverage sequencing (57.00% and 19.10%). Finally, only four samples were used for the molecular characterization of the Spanish outbreak. 

A total of 148 WNV whole genomes were found in the GenBank repository (listed in Table S1). All these genomes were downloaded and aligned, together with the four Spanish WNV sequences, using the MAFFT program (see Materials and Methods). 

### 3.2. Phylogenetic Analysis 

**Figure 2:**
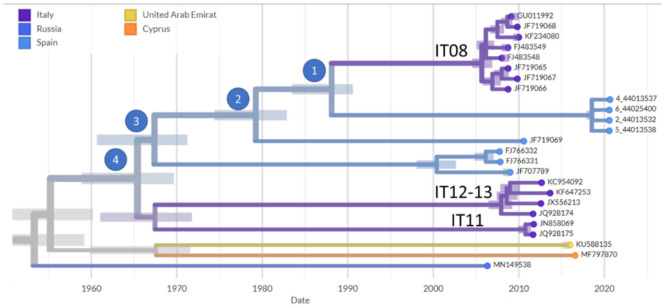
Sequences of the Spanish 2020 WNV outbreak (pale blue); the closest relatives from previous outbreaks in Italy IT08, IT11, IT12-13 (dark blue); and the sequence JF719069 from a lethal equine case in Andalusia (Spain) in 2010. Other Spanish outbreaks were: JF707789, from a mosquito in Huelva, and FJ766331 and FJ766332, from a golden eagle in Toledo [14]. Other related outbreaks from the Mediterranean region (Cyprus MF797870) [13], or adjacent locations (United Arab Emirates KU588135 and Russia MN149538) are also included. Confidence intervals for the times at which the branching points occurred are marked with bars. Branching points are labeled for the discussion.

A phylogenetic tree including the four Spanish sequences and the 148 WNV sequences listed in Table S1 was reconstructed as described in Materials and Methods. Figure 2 shows a detail of the phylogenetic tree including the current Spanish outbreak and some of the closest WNV from previous outbreaks. The closest relative outbreak sequenced was reported in Italy in 2008 (IT08) [14]. Both outbreaks share a common ancestor between 1984 and 1991 (branching point 1 in Figure 2), according to the LSD2 method [36]. However, the confidence interval of this this branch slightly overlaps with the previous branching point (2 in Figure 2) that took place between 1974 and 1984, which is the common ancestor of a lineage that originated an outbreak in Spain in 2010 (JF719069). Interestingly, these outbreaks are related to other relatively recent Italian outbreaks in 2011 [42] (IT11 in Figure 2) and 2012–2013 [12] (IT12-13 in Figure 2), whose ancestor dated from 1960 to 1971. 

The phylogenetic analysis confirms the initial assignment to lineage 1. Table 2 provides the estimated genetic distances among the WNV genome sequences shown in Figure 2. The genetic diversity in the current outbreak (between 4 and 8 nucleotide differences) is, likely due to its short duration, slightly lower than, but within the range of, that observed in previous outbreaks, such as the 2008 (4 to 23, with a median of 10.5, nucleotide differences) and 2012–2013 [12,42] (9 to 32 differences) Italian outbreaks.

### 3.3. Mutational Patterns in the Spanish Outbreak

While the general ratio of substitutions per site and year considering the whole phylogeny is 2.10 × 10^-4^, the ratio in the branch represented in Figure 2 is slightly higher (2.16 × 10^-4^). In particular, the lineage leading from branching point 1 (Figure 2) to the 2020 Spanish outbreak accumulated a total of 10 amino acid substitutions. Table 3 summarizes the mutations and the specific proteins or viral peptides affected. Interestingly, two residues mutated affect the Envelope E protein, which mediates both membrane fusion and the interaction of the virus with its cellular receptor, and holds the main neutralization sites recognized in the humoral immune response against the virus [45]. 

### 3.4. The SIEGA Nextstrain Server

The SIEGA Nextstrain server [41] offers an interactive view of the complete WNV phylogeny with the four WNV sequences belonging to the Spanish 2020 outbreak in the context of the rest of the viral sequences available, belonging to worldwide outbreaks, that have been sequenced in previous years (see Table S1). This resource was entirely developed as part of this project and has become a key tool of the Andalusian epidemiological surveillance system. Figure S1 shows a summarized view of the whole phylogenetic tree as displayed by the SIEGA Nextstrain server. 

## 4. Discussion

Regarding the origin of the WNV studied there are two possible scenarios based on the phylogeny: a common Mediterranean pool that diversifies and emerges at different locations, either in Spain or in Italy, or an endemic Italian viral reservoir, where the virus seems to be permanently active with continuous outbreaks every year since 2008 [11], from which it has been introduced several times into Spain. It is interesting to note that, at the time of branching point 4 (Figure 2), the WNV ancestor was probably located in Italy, although the three occurrences in Spain of a related virus (branching points 3, 2, and 1 in Figure 2) are also compatible with an endemic origin in the Mediterranean region that produces outbreaks in Italy or Spain. 

**Figure 3:**
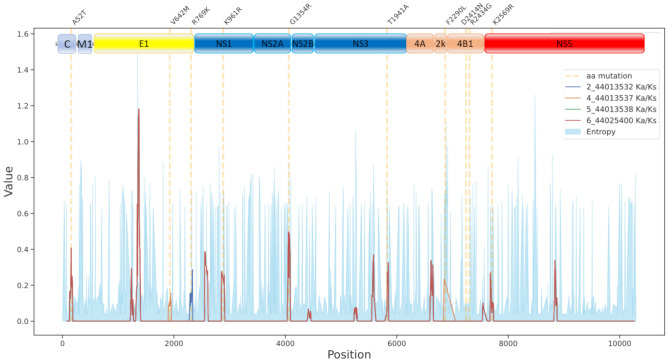
Mutational pattern observed in the Spanish outbreak with respect to its previous ancestor (branching point 1 in Figure 2) along the genome sequence. The entropy plot indicates the background variability observed in the genome of the 152 WNV sequences included in the phylogeny.

The duration of the outbreak was shorter than other recent ones (e.g., the 2008 and 2011–2012 Italian outbreaks). Similarly, when compared to other recent outbreaks, the branches corresponding to the studied viruses in the phylogeny are shorter (Figure 2) and the intra-outbreak genetic distances are smaller (Table 2). However, it is worth mentioning the mutational pattern observed in the viral sequences of the Spanish 2020 outbreak studied. Two of the viruses (4_44013537 and 2_44013532) have mutations in envelope protein E (Table 3, Figure 3), which binds to host cell surface receptors and consequently fosters fusion between viral and cellular membranes [45]. These two mutations occur in regions with low entropy (Figure 3), that is, with a relatively high conservation level. Moreover, one of these mutations, V642M, occurred in the sequence of 4_44013537 in a region with a high value of non-synonymous to synonymous ratio, suggesting some positive selection for any evolutionary advantage. The other mutation, R769K, occurs close to the cleavage site of the protein, which is only 18 amino acids apart [46]. As demonstrated in the current SARS-CoV-2 pandemic, mutations in the protein that mediates the entry of the virus into the host cell can account for increased viral transmissibility [47], infectivity [48], and even mortality of the infection [49]. The WNV Meningoencephalitis risk assessment, carried out in collaboration with the Spanish Ministry of Health [50], considers the risk of transmission in Andalusia moderate, although with high impact due to the severe neuroinvasive disease and deaths associated [18]. Other mutations shared by all the WNV sequenced are A52T—in the Capsid protein C—which forms the nucleocapsid and occurs within a predicted homodimerization domain [51], T1941A—in the NS3 protein—that occurs within a helicase C-terminal domain, according to the UniProtKB feature viewer [52], and K2569R—in the RNA-directed RNA polymerase—that occurs in a predicted RNA binding motif (mRNA cap 0 and cap 1 methyltransferase, according to Prosite [53], entry PS51591). The rest of the mutations (Table 3) do not seem to affect any specific identified domain of any protein. 

After the outbreak declaration, several measures were taken, which included the reinforcement of the active epidemiological surveillance of lymphocytic meningoencephalitis human cases and passive and active surveillance activities in horses, birds, and *Culex* mosquitoes in the areas defined as risk zones. Prevention campaigns were carried out in the affected areas by informing inhabitants of the recommendations for individual protection against mosquito bites. The West Nile Encephalitis Surveillance Plan in Spain, of the Ministry of Agriculture, Fisheries, and Food [50], which is responsible for entomological, ornithological, and equine surveillance during the vector activity season (April to November), discovered an especially high vector activity in the affected area, with an abundant presence of *Culex perexiguus* in rice-growing areas and *Culex pipiens* in urban areas. In fact, a total of 125 equine foci were declared by the Plan (58 in the province of Seville, 49 in Cádiz, and the rest in other provinces) since August 10, 2020 [18], reinforcing the importance of surveillance plans and supporting the establishment of One Health, which led to the implementation of the Comprehensive Vector Surveillance and Control Program for West Nile Fever in Andalusia [54]. In addition, it is necessary to support genomics-informed, real-time, global pathogen surveillance approaches in the control of zoonosis [55], such as the SIEGA initiative [56]. One of the key tools to support this surveillance is the WNV Nextstrain Epidemiological Local Server [41].

## Figures and Tables

**Table 1:** WNV samples studied and some statistics on the viral sequencing process. Samples came from Hospital Virgen del Rocío (HUVR), Seville, and Hospital Puerto Real (HPR), Cádiz. Depth refers to the mean number of reads covering each position. High-confidence variants (HCV) refer to the variants unambiguously identified by the calling algorithm of the nf-core/viralrecon pipeline used, and the last column refers to the percentage of the viral genome covered in the sequencing process.

Date	Code	Hospital	Sample	PCR	Culture	SAMPLE_ID	Depth	HCV	Coverage
13 August 2020	44013531	HUVR	Urine	Positive	No	1_44013531	835x	351	57.00%
13 August 2020	44013532	HUVR	Urine	Positive	No	2_44013532	1190x	461	96.10%
2013 Au-gust 2020	44013536	HUVR	Urine	Positive	No	3_44013536	465x	88	19.10%
2013 Au-gust 2020	44013537	HUVR	Urine	Positive	No	4_44013537	1043x	420	79.70%
2013 Au-gust 2020	44013538	HUVR	Urine	Positive	Negative	5_44013538	1205x	453	94.50%
9 September 2020	44025400	HPR	CSF	Positive	Negative	-			
9 September 2020	44025400	HPR	Urine	Positive	Positive	6_44025400	1145x	451	94.70%

**Table 2:** Genetic distances observed among the WNV of the current outbreak and the closest sequences in the phylogeny in terms of nucleotide differences. IT08, IT12-13, and IT11 refer, respectively, to the 2008, 2012–2013, and 2011 Italian outbreaks, with several sequences. The values are average distances between all the sequences in the cluster and the compared sequence.

	2_44013532	4_44013537	5_44013538	6_44025400	FJ766331	FJ766332	JF707789	JF719069	KU588135	MF797870	MN149538	IT08^1^	IT12-1^32^	IT11^3^
2_44013532	0.0	8.0	5.0	5.0	226.0	224.0	240.0	182.0	330.0	293.0	252.0	136.0	238.5	248.0
4_44013537	8.0	0.0	8.0	7.0	209.0	207.0	222.0	165.0	302.0	265.0	234.0	127.0	219.5	224.0
5_44013538	5.0	8.0	0.0	4.0	221.0	219.0	235.0	178.0	320.0	289.0	250.0	133.0	232.5	244.0
6_44025400	5.0	7.0	4.0	0.0	221.0	219.0	235.0	178.0	322.0	289.0	250.0	133.0	233.5	242.0
FJ766331	226.0	209.0	221.0	221.0	0.0	6.0	37.0	182.0	268.0	243.0	185.0	179.0	174.0	192.0
FJ766332	224.0	207.0	219.0	219.0	6.0	0.0	35.0	180.0	266.0	241.0	185.0	177.0	172.0	190.0
JF707789	240.0	222.0	235.0	235.0	37.0	35.0	0.0	197.0	277.0	260.0	204.0	193.5	190.0	208.5
JF719069	182.0	165.0	178.0	178.0	182.0	180.0	197.0	0.0	298.0	250.0	219.0	137.0	197.0	202.0
KU588135	330.0	302.0	320.0	322.0	268.0	266.0	277.0	298.0	0.0	246.0	258.0	295.0	277.5	298.0
MF797870	293.0	265.0	289.0	289.0	243.0	241.0	260.0	250.0	246.0	0.0	210.0	258.5	233.5	260.0
MN149538	252.0	234.0	250.0	250.0	185.0	185.0	204.0	219.0	258.0	210.0	0.0	216.0	197.5	220.5
IT08 ^1^	136.0	127.0	133.0	133.0	179.0	177.0	193.5	137.0	295.0	258.5	216.0	10.5	198.0	206.3
IT12-13 ^2^	238.5	219.5	232.5	233.5	174.0	172.0	190.0	197.0	277.5	233.5	197.5	198.0	9.75	192.3
IT11 ^3^	248.0	224.0	244.0	242.0	192.0	190.0	208.5	202.0	298.0	260.0	220.5	206.3	192.25	2.0

^1^ Sequences GU011992, JF719068, KF234080, FJ483549, FJ483548, JF719065, JF719067, and JF719066, from the 2008–2009 Italian outbreak [43]. ^2^ Sequences KC954092, KF647253, JX556213, and JQ928174 from the 2012 and 2013 Italian outbreaks [12,42]. ^3^ Sequences JN858069 and JQ928175 from the 2011 Italian outbreak [44].

**Table 3:** Amino acid mutations fixed in the lineage leading to the 2020 Spanish outbreak studied.

Mutation	Protein	Function
A52T	Capsid protein C	Form the nucleocapsid
V642M	Envelope E	Binding host cell surface and mediates membranes fusion
R769K	Envelope E	Binding host cell surface and mediates membranes fusion
K961R	NS1	Role in early RNA replication
G1354R	NS2A	Component of the viral RNA replication complex
T1941A	NS3	In association with NS2B, performs its autocleavage
F2290L	2k	Signal peptide for NS4B
D2414N	NS4B	Induces the formation of ER-derived membrane vesicles
R2434G	NS4B	Induces the formation of ER-derived membrane vesicles
K2569R	NS5	RNA-directed RNA polymerase

## Data Availability

The sequences of the four WNV presented here are available in the European Nucleotide Archive (ENA) database under the project identifier PRJEB43037.

## Supplementary Materials

The following are available online at https://www.mdpi.com/article/10.3390/v13050836/s1, Table S1: List of WNV whole genomes available in GenBank, with their IDs, isolation dates, and places, and publication references when available. Table S2: Matrix of genetic distances (as the number of nucleotide changes between pairs of sequences) estimated among all the WNV whole genomes in Table S1. Figure S1: Screenshot of the local Nextstrain server with all the WNVs from Table S1 and the Spanish sequences of the 2020 outbreak reported here. The Spanish 2020 outbreak and two previous outbreaks (2010 and 2007) are marked in the figure.
