# Supplementary material for: Phylogenetic Analysis of the 2020 West Nile Virus (WNV) Outbreak in Andalusia (Spain)
Source: Viruses. 2021 May 5;13(5):836. doi: 10.3390/v13050836 (PMC8148183; doi:10.3390/v13050836)
Supplement: Supplementary file 1 [file viruses-13-00836-s001.zip › Figure S1.R2.pdf]

Phylogenetic analysis of the 2020 West Nile virus (WNV) outbreak in Andalusia (Spain)

Carlos S. Casimiro-Soriguer[1,2,+], Javier Perez-Florido[1,2,+], Jose L. Fernandez-Rueda[1], Irene Pedrosa-Corral[3,4], Vicente Guillot-Sulay[3,4], Nicola Lorusso[5], Luis Javier Martinez-Gonzalez[6], Jose M. Navarro-Marí[3,4], Joaquin Dopazo[1,2,7,8,*], Sara Sanbonmatsu-Gámez[3,4*]

[1] Clinical Bioinformatics Area. Fundación Progreso y Salud (FPS). Hospital Virgen del Rocio. 41013. Sevilla. Spain;1; e-mail@e-mail.com

[2] Computational Systems Medicine, Institute of Biomedicine of Seville (IBIS), Hospital Virgen del Rocio. 41013. Sevilla. Spain ;

[3] Laboratorio de Referencia de Virus de Andalucía. Servicio de Microbiología. Hospital Virgen de las Nieves, Granada. Spain

[4] Instituto de investigación biosanitaria, ibs.GRANADA. Granada.Spain

[5] Dirección General de Salud Pública y Ordenación Farmacéutica. Consejería de Salud y Familias. Junta de Andalucía;

[6] GENYO. Centre for Genomics and Oncological Research: Pfizer - University of Granada - Andalusian Regional Government. Granada.Spain;

[7] Bioinformatics in Rare Diseases (BiER). Centro de Investigación Biomédica en Red de Enfermedades Raras (CIBERER). FPS. Hospital Virgen del Rocio. 41013. Sevilla. Spain;

[8] Functional Genomics Node (INB). FPS. Hospital Virgen del Rocio. 41013 Sevilla. Spain.

+ Equal contributions

* Correspondence: JD: joaquin.dopazo@juntadeandalucia.es, SB-G: saral.sanbonmatsu.sspa@juntadeandalucia.es

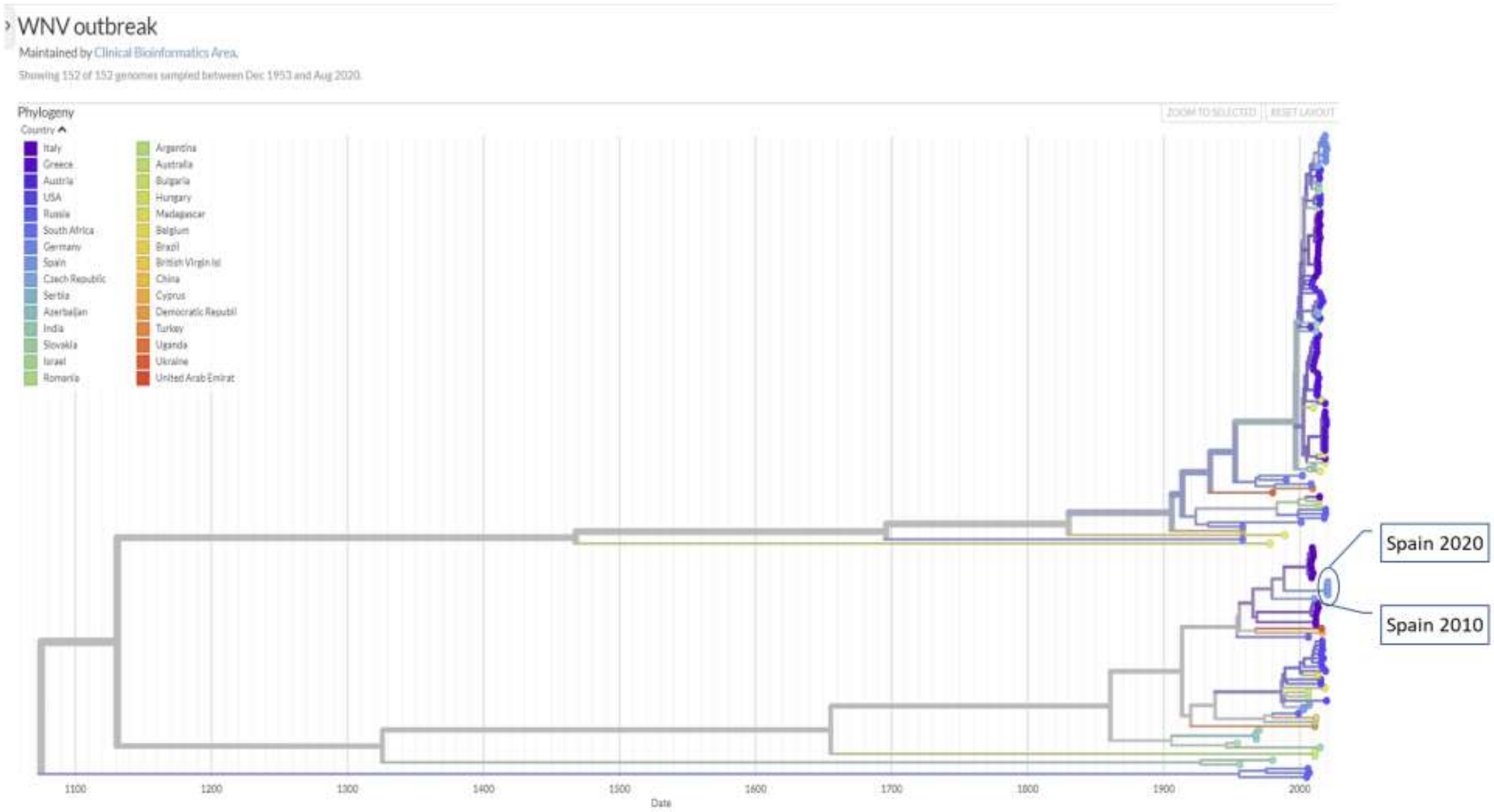

Figure S1. Screenshot of the local Nextstrain server with all the WNVs from Table S1 and the Spanish sequences of the 2020 outbreak, reported here. The Spanish 2020 outbreak and previous outbreaks (2010) are marked in the figure. The tree is available at: http://nextstrain.clinbioinfosspa.es/wnv
